# Novel drugs affecting diabetic peripheral neuropathy

**DOI:** 10.22038/IJBMS.2024.75367.16334

**Published:** 2024

**Authors:** Dalia O. Saleh, Ahmed A. Sedik

**Affiliations:** 1 Pharmacology Department, Medical Research and Clinical Studies Institute, National Research Centre, 12622, Egypt

**Keywords:** Diabetic peripheral- neuropathy, Hyperglycemia, Molecular mechanism, Neuropathic pain, Quality of life, Therapeutic targets

## Abstract

Diabetic peripheral neuropathy (DPN) poses a significant threat, affecting half of the global diabetic population and leading to severe complications, including pain, impaired mobility, and potential amputation. The delayed manifestation of diabetic neuropathy (DN) makes early diagnosis challenging, contributing to its debilitating impact on individuals with diabetes mellitus (DM). This review examines the multifaceted nature of DPN, focusing on the intricate interplay between oxidative stress, metabolic pathways, and the resulting neuronal damage. It delves into the challenges of diagnosing DN, emphasizing the critical role played by hyperglycemia in triggering these cascading effects. Furthermore, the study explores the limitations of current neuropathic pain drugs, prompting an investigation into a myriad of pharmaceutical agents tested in both human and animal trials over the past decade. The methodology scrutinizes these agents for their potential to provide symptomatic relief for DPN. The investigation reveals promising results from various pharmaceutical agents tested for DPN relief, showcasing their efficacy in ameliorating symptoms. However, a notable gap persists in addressing the underlying problem of DPN. The results underscore the complexity of DPN and the challenges in developing therapies that go beyond symptomatic relief. Despite advancements in treating DPN symptoms, there remains a scarcity of options addressing the underlying problem. This review consolidates the state-of-the-art drugs designed to combat DPN, highlighting their efficacy in alleviating symptoms. Additionally, it emphasizes the need for a deeper understanding of the diverse processes and pathways involved in DPN pathogenesis.

## Introduction

Diabetic peripheral neuropathy (DPN) is a common and disabling chronic complication of diabetes, with high prevalence in type II DM patients, with about a quarter of them experiencing pain (1). DPN is tightly linked with increased mortality and leads to morbidity, and mostly leads to two main clinical concerns: diabetic foot ulceration and neuropathic pain (2). A complicated interplay of risk factors and patient behavior leads to diabetic foot ulceration, but sensory loss related to DPN is frequently the underlying cause (3). Furthermore, painful neuropathic symptoms affect up to 50% of DPN patients (4). These painful sensations are commonly harsh and usually lead to psychological events, the most important of which are depression, anxiety, and sleep disorders, as well as impairment of quality of life (QOL)(2). 

Furthermore, diabetes-related lower-limb problems result in distressing and serious clinical consequences such as leg amputation and ultimately death (5). Unfortunately, DPN is generally often diagnosed after permanent nerve damage has already taken place and its initial manifestation could be a diabetic foot ulcer. Typically, neuropathy is nerve damage that proceeds proximally, beginning with the longest nerves that innervate the limbs appearing as numbness, tingling, discomfort, and/or weakness beginning in the distal lower limbs (6).

Diabetes mellitus (DM) is known as the most important metabolic risk factor for neuropathy, however, treatment of hyperglycemia is not enough to avoid neuropathy in those with type II DM (7). There is strong evidence that type I DM patients with strict glycemic management have a lower risk of developing DPN. Nevertheless, in DPN, symptomatic treatments are frequently insufficient and neither glucose control nor pharmacological treatments are successful. The prevalence of DM, DPN, and foot amputations is increasing at an alarming rate. The condition must be diagnosed early and accurately so that measures may be implemented to reduce the risk of diabetic foot complications. Metabolic syndrome, which is tightly related to obesity and insulin resistance, has been implicated in the development of peripheral neuropathy independent of hyperglycemia (6, 8). However, Stino and Smith suggested that many patients diagnosed with idiopathic peripheral neuropathy may have pre-diabetic neuropathy (8).

***Classification***

Diabetic neuropathy is divided into diffuse and focal neuropathies. Diffuse neuropathies are subdivided into DPN and diabetic autonomic neuropathy.  Nerves in the extremities are typically affected by peripheral neuropathies, which affect both small and large nerve fibers. Damage to the large nerve fibers restricts the body position and movement, whereas demyelination of smaller nerve fibers in the peripheral region causes neuropathic pain dysesthesias and paresthesia (9). DPN can take several different forms, with distal symmetry diabetic sensorimotor polyneuropathy being the most prevalent type. Seventy to ninety percent of all DN instances are caused by diabetic sensorimotor polyneuropathy, which can be painful DPN (pDPN). Burning, stabbing, numbing, or deep aching pains are experienced in the periphery of pDPN due to the involvement of many neurons (10). Unfortunately, pDPN is the most common subtype of DN. It is described as “pain resulting from somatosensory system damage attributable to DM. “ The other subtypes are diabetic lumbosacral radiculoplexus neuropathy, mononeuropathy, small fiber neuropathy, and mononeuritis multiplex neuropathy (11).

***Symptoms***

Diabetic peripheral neuropathy has a variety of clinical symptoms. Both negative and positive sensory symptoms are possible. Numbness or “deadness” are negative symptoms that patients may compare to the sensation of wearing gloves or socks. Aching, tingling, burning, an “electric shock” sensation, and hypersensitivity to touch are examples of positive sensory sensations (12). The potentially severe pain associated with DPN probably leads to insomnia, anxiety, depression, and activity impairments, in addition to a reduced QOL (13). DPN motor symptoms can be proximal or distal, localized or diffused. Motor symptoms in the hands may include a lack of coordination. Patients with motor symptoms may also experience difficulties rising from a prone position, limb weakness with repeated tripping or toe scraping, with weak knees during stair climbing (14). 

***Pathophysiology***

**Figure 1:**
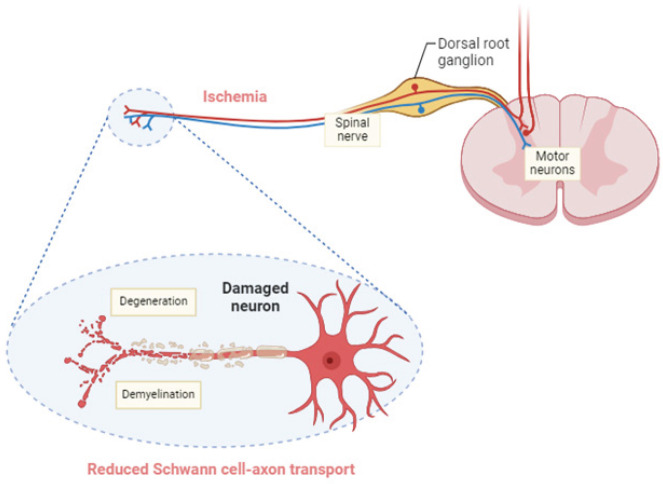
Hyperglycemia-driven neuronal damage and Schwann cell stress

The development of DPN often involves both vascular and metabolic factors. Patients with DPN frequently experience nerve fiber loss brought on by poor blood flow, which reduces nerve sensitivity and, as a result, pain perception. Compared to healthy people, these patients may also experience lower oxygen tension, vascular malformations, and hypertrophy. These effects highlight the relationship between vascular and neurostructural alterations in DPN patients (15). Hyperglycemia as well as dyslipidemia, which are the major manifestations present in diabetic patients, usually involve multiple cells in the peripheral nervous system, comprising neuronal axons, dorsal root ganglion (DRG) neurons, and Schwann cells. Disturbance in the neuronal support including the Schwann cells and the vascular system leads to neuropathy, concurrently with the direct effects of hyperglycemia on neurons (7) as presented in Figure 1.

Furthermore, reactive oxygen species (ROS) accumulation, ATP production loss, mitochondrial dysfunction, endoplasmic reticulum stress pathway activation, and advanced glycation end products (AGEs) development are among the impacts of hyperglycemia on these cells. Hyperglycemia has a significant impact on both vascular and neural tissues. ROS levels elevate even more, which in turn encourages endoplasmic reticulum stress, DNA damage, apoptosis, and the activation of pro-inflammatory signaling-all of which are mechanisms that eventually result in nerve injury (16). The sensory sensitivity and demyelination of peripheral neurons are produced by DM-induced oxidative stress. According to a rat study, metabolic flow is the main factor contributing to demyelination and the development of peripheral neuropathy (17). 

**Figure 2:**
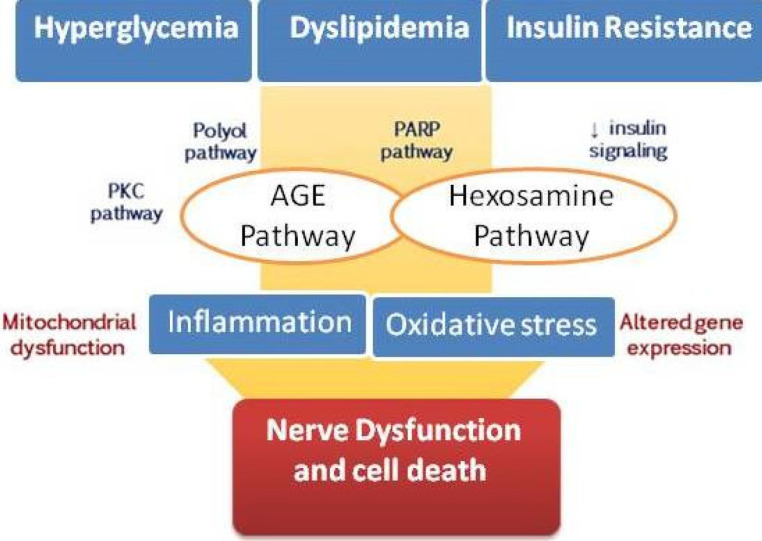
Pathways involved in diabetic neuropathy pathogenesis

Nevertheless, the pathophysiology of DPN is still not fully understood. Figure 2 shows several molecular pathways including the activation of the polyol pathway, hexosamine pathway, poly ADP-ribose polymerase (PARPs) pathways, oxidative stress, inflammation, protein kinase C activation, and the creation of AGEs, which are associated with pathogenic neural alterations and functional nerve damage (18). Recent advances indicate that the accumulation of these damaging events may result in neuronal death. Furthermore, mechanistic and pathological findings are unable to differentiate between painful- and painless-DPN (19).

***Management overview***

*Goals of therapy*

**Figure 3:**
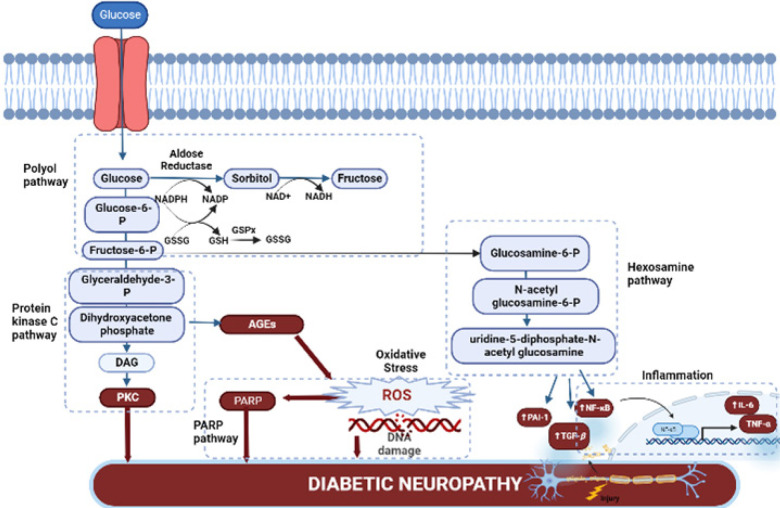
Proposed metabolic targets for the treatment strategies of diabetic peripheral neuropathy (DPN)

Consequently, there are no pharmacotherapies that can effectively modify the disease and treat the condition. Controlling risk factors for DPN and preventing and managing its consequences is the cornerstone of contemporary management. Similarly, although there are some variations between DPN that are painless and painful DPN, the precise mechanisms that cause the disease remain unknown. Painful DPN (pDPN) is not frequently treated with disease-modifying therapies; instead, treatment is mostly symptomatic as they are ineffective and poorly tolerated (20). Recent breakthroughs in the understanding of the pathophysiology of DPN have played a major role in the development of novel therapeutic agents, though still in pre-clinical trials, that may be of great use in clinical practice in the future. These potential therapeutic agents include signaling molecule inhibitors or suppressor signal activators as indicated in the pathophysiology of DPN, which has been diagrammatically presented in Figure 3. Treatment goals in DPN patients include modulation of these metabolic pathways and pain modulation, as well as enhanced glucose control. For DPN patients, several guidelines have advocated the use of pharmacological therapies, both on and off-label, to reduce pain and thus enhance QOL.

***Pharmacological agents***

**Novel therapeutic agents targeting metabolic pathways of DPN**

*Inhibition of polyol pathway*

Aldose reductase (AR) along with sorbitol dehydrogenase are the two primary enzymes in the polyol pathway, and they are in charge of the metabolism of excess glucose (21). This route consumes extra glucose in a hyperglycemic condition, increasing NADPH levels and creating reductive stress (22)(Figure 3). This stress as well as the mitochondrial dysfunction impairs the activity of the Schwann cells, which compromises myelination, results in aberrant neurotrophic support for the axon, and ultimately results in a loss of axon function (23). Moreover, the structural degradation of nerves effects on axon-glia dysfunction and lower nerve conduction velocity (NCV) are also detected. It also causes the down-regulation of the glutathione reduction pathway, which builds up ROS and thus aggravates nerve injury by causing NO-mediated vasodilation (24). 

Epalrestat, a carboxylic derivative, is a reversible aldose reductase inhibitor (ARI) thus inhibiting the polyol pathway which has been verified to be effective against DPN. Clinical studies revealed marked amelioration in the spontaneous pain in the distal limbs of DM patients (25). Epalrestat has been found to be beneficial in protecting against nerve damage induced by hyperglycemia, with an acceptable safety profile (21). However, Chalk *et al*. demonstrated that Epalrestat induces hepatic oxidative stress and inflammation and stimulates liver fibrogenesis. Therefore, caution should be exercised during the therapy (26). 

**Figure 4:**
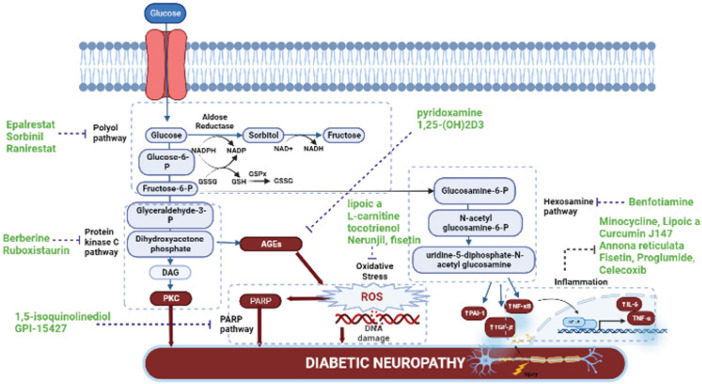
Proposed novel therapeutic agents targeting different metabolic pathways of diabetic peripheral neuropathy (DPN)

Although other ARIs, *viz*., fidarestat, ponalrestat, zopolrestat, and lidorestat, have been used to treat diabetic complications, their side effects prevent them from having the desired results. In addition, some other ARIs such as sorbinil (27) and ranirestat (28) have been advanced into the late stage of clinical trials and found to be safe for human use due to the positive results in improving NCV, sensory perception, and nerve fiber density in the patient suffering from diabetic polyneuropathy. Although several ARIs have been approved for use in DPN in Japan and other countries, most regulatory agencies have not approved any specific ARIs to alter the course of DPN (29)(Figure 4).

*Hexosamine pathway blockage*

In type II DM, insulin resistance and hyperinsulinemia are tightly related to glutamine fructose-6-phosphate amidotransferase.Uridine-5-diphosphate-N-acetyl glucosamine, the end-product of this pathway, causes gene transcription factor to elevate which stimulates the transforming growth factor beta (TGF-*β*) and plasminogen activator inhibitor-1 (PAI-1) responsible for injuring the endothelial cells and stimulating smooth muscle cell division (Figure 3). This further results in microvascular problems, including DN, which harms the blood vessels that supply the nerves with blood (30, 31).

Benfotiamine, a lipid-soluble derivate of thiamine (vitamin B1) with a high bioavailability, has shown a great reduction in the AGEs by hindering glucose metabolism through hexosamine pathway blockage (32). In 2022, a study was conducted to evaluate the efficacy and safety of benfotiamine in DPN patients. A benfotiamine dose of 300 mg two times a day was accompanied by neuropathic symptom improvement after 5 weeks with no severe adverse events (33)(Figure 4). Moreover, it was found that co-administration of benfotiamine and alpha lipoic acid, an antioxidant, exhibited greater effectiveness than the monotherapy of the drugs (34).

*Inhibiting protein kinase C pathway*

Several studies have confirmed the involvement of protein kinase C (PKC) in DN (35). Glyceraldehyde-3-phosphate is transformed into dihydroxyacetone as part of the PKC pathway, which is then transformed into glycerol-3-phosphate and finally into diacylglycerol (DAG) (Figure 3). DAG and AGEs up-regulate PKC, causing normalization of sciatic NCV and nerve blood flow via down-regulation of Na^+/^K^+^ ATPase (6). 

By down-regulating the PKC pathway in diabetic rats, the hyperexcitability of C-fiber was reduced (36). Berberine is a plant alkaloid that was found to ameliorate DPN in rats by modulating PKC as well as inhibiting TNF-α (37). Ruboxistaurin, a PKC inhibitor, has been extensively studied in DPN with promising results (38). Moreover, ruboxistaurin has demonstrated *in vitro *and* in vivo* improvement in blood flow related to hyperglycemia and has potential use as a therapy for diabetic retinopathy (39)(Figure 4).

*Poly ADP-ribose polymerase (PARPs) deactivation*

Under normal conditions, poly ADP-ribose polymerase (PARPs) is involved in DNA repair and apoptosis induction. In DM, accumulation of PARPs causes tissue damage (Figure 3). Hyperglycemia leads to accumulation of ROS and reactive nitrogen species, which breaks DNA strands. The resulting overexpression of PARPs results in damaging the blood arteries that supply the nerves (40). Additionally, PARP activation is linked to energy loss processes in diabetic animals as well as nerve conduction deficits in sensory and motor nerves, dysfunction of the neurovascular system, gene expression, altered transcriptional control, and dysfunction of the neurovascular system (41). 

Interestingly, a PARPs inhibitor, 1,5-isoquinolinediol alleviates experimental diabetic sensory neuropathy (42). Similarly, GPI-15427, which is another PARP inhibitor, resulted in alleviation of DPN symptoms as well as the reduction of intra-epidermal nerve fiber degeneration after oral administration in rodent models of progressive type I DM. The outcome of the study has shown that there is a great need for the development of potent and low-toxicity PARPs (43)(Figure 4).

*Advanced glycation end products reduction*

Advanced glycation end products (AGEs) along with their receptor for advanced glycation end products (RAGE) accumulate as a result of non-enzymatic reactions involving glucose and other saccharides that change the composition and functionality of proteins and lipids (26)(Figure 3). Much research on AGEs has demonstrated that they harm blood vessels. Patients with type I DM were reported to have elevated AGE/RAGE levels (18). It has been established that endothelium and Schwann cells contain AGE/RAGE. By elevating the p65 subunit of NF-kB, which causes inflammation and damage in myelinated neurons, high levels of AGEs cause DN. Additionally, AGEs are linked to the triggering of Schwann cell death (44, 45). Therefore, in an attempt to alleviate DPN, recent studies have targeted AGEs. Xu *et al*. have shown that interleukin 10 (IL-10) which is an anti-inflammatory cytokine was reported to have a beneficial effect on the Schwann cells against AGE via regulating the NF-*κ*B pathway (46). 

Furthermore, researchers have reported that treatment of diabetic rats with pyridoxamine temporarily relieved their DPN symptoms via its ability to block the RAGE- NF-κB/ERK signaling pathway (47). Similarly, it has been observed that co-administration of AGEs and 1,25-(OH)2D3, active form of vitamin D3, to Schwann cells leads to suppression of apoptosis, induced by AGEs via the NF-*κ*B pathway (44, 48)(Figure 4). Other studies have also reported that DPN symptoms are relieved by injection of vitamin D intramuscularly (49). Therefore, by counteracting the detrimental consequences of AGEs, vitamin D treatment boosts the neuroprotective effect of Schwann cells (49). 

*Oxidative stress amelioration*

Oxidative stress is a crucial component of DN, which is primarily brought on by free radicals produced when excess glucose is diverted to the polyol pathway, hexosamine pathway, PKC pathway, and AGE/RAGE interaction (48). These elements work together to intensify intracellular imbalanced redox homeostasis, and abnormal protein, lipid, and DNA alterations, which cause mitochondrial damage and excessive ROS generation (Figure 3). Peripheral nervous system damage results from the loss of sensory neurons, myelinated axons, and Schwann cells in the DRG. Additionally, insufficient mitochondrial energy production worsens axonal damage in DN by impairing information transmission down the axons. While NF-κB is associated with the induction of an inflammatory response, Nrf2 is a transcription factor that is activated by the redox status of the environment and controls the antioxidant system (50-52). The control of both of these factors is coordinated in healthy cells to keep the redox balance, but in DN, this balance is disrupted (53).

In the past few decades, numerous agents targeting oxidative-nitrosative stress have been evaluated in an attempt to overcome DPN. The most important of which is alpha lipoic acid, a widely tested drug for DN, which has been shown to elevate the vital endogenous antioxidant, reduced glutathione (54). In clinical research, it was discovered that 600 mg of alpha lipoic acid improved neuropathic abnormalities such as hyperalgesia, numbness, and paresthesia (55). Furthermore, several mechanisms, including antioxidant, anti-apoptotic, and cytoprotective activities, have been shown to contribute to the relief of DPN symptoms by acetyl L-carnitine (56).

In diabetic rats, tocotrienol and insulin were found to reverse DPN by regulating oxidative-nitrosative stress, caspase 3, and pro-inflammatory cytokines (57). Moreover, berberine has been proposed to reduce the boosted oxidative stress and inflammation in the neurons, hence reducing DM and DPN (58). Likewise, Nerunjil (*Tribulus terrestris*) has been reported to improve the pain threshold in DPN via modulating oxidative stress and thus the inflammatory responses (59). On the other hand, fisetin is a neuroprotective in experimental diabetic neuropathy via modulating both Nrf2 and NF-κB pathways (60). 

In addition, *Rosmarinus officinalis L.* reduces caspase and the Bax/Bcl-2 ratio, important signaling molecules that trigger apoptosis, and has anti-nociceptive and neuroprotective effects in diabetic rats via radical-scavenging properties. In STZ-induced diabetic rats, a significant effect was detected as decreased thermal hyperalgesia (61). In diabetic animal models, the use of kaempferol, derived from the *Eruca sativa* plant, ameliorates DM-induced nerve damage by attenuating oxidative stress (62)(Figure 4).

*Inflammation regulation*

Proinflammatory cytokines play a crucial role in the pathogenic signals of DN. In peripheral nerves and the spinal cord of sedentary STZ-induced diabetic rats, Yu-Wen *et al*. found noticeably higher levels of IL-6 and TNF-α and how these cytokines contribute to DPN (63). It is widely known that TNF-α is inversely correlated with the intensity of pain (64, 65)**(**Figure 3**)**. Patients with DPN showed an elevated level of IL-10 due to the compensatory mechanism being activated (66). Minocycline reduces DPN in STZ-treated rats and enhances the analgesic properties of morphine via increasing the production of IL-10, IL-2, and IL-1α, thus preventing pancreatic beta cell necrosis and inhibiting PARPs (67, 68). Another potent possibility for a medication that reverses touch-triggered allodynia is the curcumin derivative J147, a novel derivative of curcumin for the treatment of DN, boosts the AMP kinase pathway and suppresses TNF-α and other neuroinflammatory indicators that cause neurodegeneration (69). Furthermore, a bioactive fraction of *Annona reticulata* bark or *Ziziphus jujuba* root bark attenuates DN by blocking the NF-κB inflammatory cascade (70). Likewise, researchers have reported the NF-κB cascade and transient potential vanilloid receptor type 1 channel (TRPV1) expression in diabetic rats are modulated by alpha lipoic acid, which is also reported to ameliorate DPN (71). By modulating the transcription factor Nrf2 and NF-κB regulation, fisetin has been demonstrated to impart neuroprotection in experimental diabetic neuropathy (72).

It has been also observed that COX-2, which is normally dormant, becomes active in response to hyperglycemia, oxidative stress, PKC activation, and inflammatory cytokines (73). The selective COX-2 antagonist celecoxib is well known for reducing allodynia and hyperalgesia in diabetic rats via regulating opioid receptors or voltage-gated sodium and potassium ion channels. Proglumide, a nonselective cholecystokinin inhibitor receptor, was combined with celecoxib and this had a considerable positive impact on the diabetic rats’ painful sensation (74). Another COX-2 antagonist, meloxicam, is also advocated for treating allodynia in diabetic animals (75). Likewise, COX-2 inhibitors (SC-58125 and NS-398) when administered intrathecally produced a marked anti-hyperalgesic effect in diabetic animals (76)(Figure 4).

*Mitogen-activated protein kinases inhibitors*

C-Jun N-terminal kinase (JNK), extracellular signal-related kinase, and p38 are the three types of mitogen-activated kinases that are each involved in signal transduction. While JNK and p38 promote neuronal death, ERK domains 1 and 2 are linked to brain survival. These three are up-regulated, which causes neuropathic pain. In diabetic rats, JNK down-regulation leads to neural regeneration while JNK overexpression phosphorylates neurofilaments (41). It has been demonstrated that MAPK inhibitor: U0126 and p38 MAPK inhibitors: SB203580 and SD-282 as well as JNK inhibitor: SP600125 have major roles in repairing mechanical allodynia and hyperalgesia in animal models of DM (77, 78). Additionally, it was noted that in the experimental model of DN in rats, the neuroprotective action of berberine is mediated by the MAPK signaling system (79) as well as its ability to modify PKC and inhibit TNF-α in DPN (37).

*Pyruvate dehydrogenase kinases (PDKs) inhibition*

In glycolysis, glucose is converted to pyruvate, which is subsequently transported into the mitochondria where it undergoes oxidative decarboxylation to create acetyl CoA. This process is regulated by the mitochondrial enzyme pyruvate dehydrogenase complex (PDC). The PDC may be phosphorylated by pyruvate dehydrogenase kinases (PDKs), thus inhibiting it and the excess pyruvate is converted to lactic acid (80). The increase in lactic acid caused by stimulation of PDKs interposes the pathogenesis of DPN and ultimately leads to central sensitization and pain hypersensitivity. It has been demonstrated that genetically eliminating PDK2 and PDK4 reduced DPN in streptozotocin (STZ)-induced diabetic rats and the researchers concluded that the glucose-PDK2/4-PDC-lactate pathway in the DRG may be a possible pharmaceutical therapeutic target for DPN (81). In 2022, it has been proposed that dichloroacetate, a PDK inhibitor, ameliorates type II DM (82) which suggested being a potential treatment for DPN.

*Long non-protein coding RNA*

The long non-protein coding RNA NONRATT021972 has been shown to be elevated in DPN as well as in the etiology of nervous system illnesses. BzATP-activated currents are noticeably higher than in control rats in the DRG SGCs of diabetic rats. When the impact of small interfering RNA (siRNA) for NONRATT021972 was evaluated in 2016, it was discovered that injection of NONRATT021972 siRNA intravenously down-regulated P2X7, TNF-α and glial fibrillary acidic protein (GFAP). Furthermore, the NONRATT021972 siRNA therapy decreased the ATP-activated currents and the DN pain feelings that followed (83). In 2020, uc.48+ siRNA and BC168687 siRNA were reported to decrease the DPN symptoms by reducing the pro-inflammatory cytokine levels (84).

*Micro-RNAs and stem cell therapy*

It is known that micro-RNA-146a controls a number of immunological disorders. MiR-146a is markedly down-regulated in type II diabetic mice, and systemic injection of miR-146a to these animals raises miR-146a levels in plasma and sciatic nerve tissue. In the sciatic nerve tissue, miR146a considerably enhanced the motor and sensory NCVs and regional blood flow by suppressing several pro-inflammatory genes and downstream cytokines (85). Exosomes produced by mesenchymal stromal cells were also demonstrated to improve NCV in DPN and reduce neurovascular dysfunction in rats (86). Mechanistic stimulation testing and radiant heat assays were dramatically improved, along with a reduction in the serum levels of various pro-inflammatory cytokines when mesenchymal stem cells were treated with anti-inflammatory activities in diabetic mice (87).

**Novel therapeutic agents targeting the neuropathic pain of DPN**

Although the pathophysiology of the neuropathic pain associated with DPN is not completely understood, it is likely caused by both central and peripheral pathways, which makes the treatment of pDPN more challenging (88). Typically, a treatment is considered effective in clinical trials when there is a reduction in pain level by a minimum of 50% along with some additional positive benefits on sleep, exhaustion, depression, and QOL (88, 89). Pregabalin and duloxetine, which are pharmacological drugs, are recognized by the US Food and Drug Administration (FDA) as the first-line treatments for pain associated with DPN (90). 

Various other agents are in clinical use for symptomatic relief, including antidepressants, anti-convulsants, opioids as well as topical agents, or a combination of all these classes of drugs (90). In randomized controlled trials, it has been demonstrated that these medications, either alone or in combination, diminish neuropathic pain in comparison to placebo; nonetheless, the majority of patients still get insufficient pain relief (91). Therefore, there is a great urge for additional therapeutic agents to defeat the neuropathic pain associated with DPN.

*Antidepressants*

Studies have proposed that DPN is accompanied by an unbalanced neuronal release of NE and 5-HT (92). Consequently, serotonin-norepinephrine reuptake inhibitors (SNRIs) are a promising class of antidepressants for DPN treatment (93). Tricyclic antidepressants (TCAs), such as amitriptyline and nortriptyline, have also exhibited positive results in DPN patients (94) and are considered a first-line therapy for DPN by many clinicians. However, TCAs use is constrained due to their side effects’ prevalence and severity, which might involve drowsiness, cardiac arrhythmias, and postural hypotension. Typically, SNRIs are more well-tolerated than TCAs (95). 

Dual serotonin and norepinephrine reuptake inhibitors (SNRI) such as duloxetine and venlafaxine have more balanced nor-adrenergic to serotonergic effects than TCA and SSRI. Duloxetine is considered the first-line drug for DPN (96). Duloxetine, an SNRI, has been recognized to be the first FDA-approved drug for the treatment of the DPN associated neuropathic pain (97). Although the precise mechanism underlying the drug’s ability to reduce central pain is unclear, it is thought to be connected to serotonergic and noradrenergic potentiation in the central nervous system (CNS). It is well known that blocking NE reuptake in particular reduces neuropathic pain (98). There were no substantial differences in the 24-hour pain severity scale between duloxetine and pregabalin in randomized, double-blind, placebo-controlled studies evaluating DPN patients (99). Kaur *et al.* compared duloxetine with amitriptyline in treating DPN patients in a randomized study, where both treatments achieved a marked improvement in pain (100). 

Ammoxetine is a novel, potent next-generation duloxetine analog. It is now being researched in animal models of different types of pain. Ammoxetine has been demonstrated to reduce microglial activation and block the release of p-p38 and JNK pathways, which are known to cause inflammation, neuropathic pain, and fibromyalgia-related pain (101). 

Moreover, desvenlafaxine, a more recent SNRI congener that is considered to be the most potent metabolite of the parent molecule venlafaxine, has recently been studied in patients with pDPN. Studies have shown that desvenlafaxine at daily doses of 200 and 400 mg is efficient in reducing pain and enhancing activity in a Phase III clinical trial (102). 

Recently, LPM580098, 1-[2-(dimethylamino)-1-(4-phenoxyphenyl) ethyl] cyclohexanol, is a novel triple reuptake inhibitor of 5-HT, NE as well as dopamine has shown ameliorative properties against neuropathic pain. It has been demonstrated that LPM580098 effectively reduces neuropathic pain without causing unwanted drowsiness or somnolence (103). 

On the other hand, TCAs have limited therapeutic efficacy for neuropathic pain, yet used. TCAs such as amitriptyline are believed to inhibit the reuptake of 5-HT and NE (95) as well as antagonize the *N*-methyl-d-aspartate (NMDA) receptors, thus reducing hyperalgesia and allodynia (104). Amitriptyline has been used as a first-line therapy for DPN since 1977 (105). However, the use of amitriptyline is limited due to its potential major side effects, such as cardiac arrhythmias and orthostatic hypotension, related to its anticholinergic effects. Moreover, amitriptyline also did not succeed in demonstrating superiority over pregabalin as well as gabapentin in relieving DPN pain (106). Similarly, desipramine has an analgesic mechanism of action same as amitriptyline in DPN patients, 5-HT/NE reuptake inhibition, and NMDA receptor blockage (108). Desipramine, in contrast to amitriptyline, has a low affinity for muscarinic (cholinergic) receptors (107) and is hence linked to less severe anticholinergic side effects (95). Desipramine provided patients with DPN substantially more pain alleviation than a placebo (12).

*Anticonvulsants*

Anticonvulsants include two general groups: traditional agents such as carbamazepine and valproate sodium along with newer agents such as calcium channel α2-δ ligands such as pregabalin and gabapentin (93). Since the 1960s, conventional anticonvulsants have been utilized to alleviate neuropathy (108).

Pregabalin was the second medication to receive FDA approval in December 2004 for the treatment of DPN neuropathic pain, three months after duloxetine received approval for the same indication. Pregabalin is suggested as the first-line treatment for DPN in the American Academy of Neurology (AAN) guidelines due to its efficiency in lowering pain and pain-related sleep disruption (109). Pregabalin is a structure related to the primary inhibitory neurotransmitter in the CNS: gamma-aminobutyric acid (GABA)(110). The binding of the α2-δ subunit of voltage-gated calcium channels is tightly correlated to its anti-nociceptive activity. Additionally, it has been demonstrated that pregabalin significantly decreased DPN-related discomfort and pain-related sleep disruption. Preclinical data supported a potential mechanism of action that would involve lowering abnormal neuronal excitability by reducing GABA neurotransmitter release (111). It is used as an adjuvant therapy for these patients (112). 

Gabapentin is not yet approved by the FDA for the treatment of DPN sufferers (113). However, as a less expensive alternative to pregabalin, published treatment guidelines have encouraged the usage of gabapentin for this indication (114, 115). Like pregabalin, gabapentin is structurally related to GABA and shares an identical therapeutic target (113). Animal studies indicate that this drug’s pain-modulating properties may be related to the release of GABA in spinal cord pathways that regulate pain perception (116). A study assessed gabapentin in early research for the symptomatic treatment of DPN patients and found that patients receiving gabapentin experienced much less pain than those receiving a placebo (117). Gabapentin was beneficial in treating a subgroup of individuals with DPN, as evidenced by a decrease in pain intensity (118). Compared to other drugs, gabapentin showed superiority over placebo in reduction in pain, according to a recently published meta-analysis conducted in 2021 (119).

*Opioids*

This class comprises the most promising novel agents that have recently gained great acceptance for neuropathic pain, according to Rastogi and Jude (120). However, the opioids used for the treatment of DPN are controversial (121) as they may lead to tolerance, frequent dose escalation, along with hyperalgesia as a result of chronic use (122). However, the therapeutic use of these medications should be reserved for DPN patients who cannot attain pain relief with other therapies. Consensus guidelines have indicated that continuous opioid medication may be advantageous for DPN patients, despite concerns regarding dependence (123).

Oxycodone is an opioid analgesic drug that is a substance listed on Schedule II, and its abuse potential is comparable to that of other opioid agonists. Oxycodone’s analgesic effect is thought to be involved in CNS opioid receptors for endogenous substances with opioid-like action that have been found to exist in the brain and spinal cord (124). It has been evaluated that oxycodone-controlled release as a DPN therapy provided a marked analgesic effect with opioid-related adverse events (125). However, Gaskell *et al*. have shown that there is no convincing evidence that oxycodone-controlled release is effective in treating DPN patients (118). It is therefore best reserved as add-on therapy for selective patients who are not at risk of opioid dependence and abuse (126).

Morphine sulfate is a strong, relatively selective agonist of the μ-opioid receptor. It interacts with one or more types of opioid receptors to provide its main therapeutic effect, which is analgesia in DPN patients (127). Gilron *et al.* have compared the effectiveness of combining sustained-release morphine along with gabapentin in patients with DPN. The outcome of this study showed that the mean daily pain of patients receiving the gabapentin/morphine combination is less than those receiving each drug alone (128, 129).

*Opioid-like analgesics*

Tapentadol**,** a synthetic μ-opioid receptor agonist and NE reuptake inhibitor, received FDA approval in July 2012 for DPN treatment. The third drug to have this indication approved after duloxetine and pregabalin. Tapentadol is suggested for individuals with pDPN that is severe enough to need a 24-hour opioid medication and for whom other treatment choices are insufficient (130). In clinical investigations on DPN patients, the most frequent adverse effects of tapentadol are headache, nausea, dizziness, sleepiness, constipation, and vomiting. Because tapentadol has a dual opioid/NE mode of action, its gastrointestinal side effects are typically less severe than those of ordinary opioids (12). This may make it a better option for chronic pain management. However, due to its limited effectiveness in reducing pain, safety issues, and the high risk of addiction, new guidelines do not recommend it as a first or second-line therapy (131).

Tramadol is a synthetic, centrally-acting analgesic in a sustained-release formulation. The parent drug and its metabolite appear to bind to μ-opioid receptors and there is also a mild suppression of both NE and 5-HT reuptake, which together appear to be at least two complementary mechanisms underlying its analgesic effect. Although it is not specifically approved for DPN patients, tramadol is prescribed for those with moderately to moderately severe chronic pain who require ongoing care for a significant amount of time (132). According to the report of the AAN Guidelines Committee 2022, tramadol “may be considered” for DPN treatment, but there is no sufficient information to favor it over oxycodone, morphine sulfate, or dextromethorphan (109). Moreover, the effectiveness and safety of taking tramadol and acetaminophen together for DPN were assessed. The combination reduced DPN symptoms including pain, sleep quality, mood, anxiety, and QOL; nonetheless, the trial was stopped early due to the unfavorable results (133). 

Dextromethorphan is a synthetic NMDA receptor antagonist indicated as an antitussive and expectorant (134). It has been clinically confirmed that dextromethorphan is effective at managing DPN due to its capacity to attach to NMDA receptors in the spinal cord and CNS and so prevent the production of central acute and chronic pain sensations (135). It has few side effects if used at the recommended doses (134). According to the report of the AAN Guidelines Committee 2022, dextromethorphan is “probably beneficial” in reducing DPN pain, however, there is not enough evidence to support its use as a treatment option above oxycodone, morphine sulfate, or tramadol (109).

*Cannabinoid receptor agonists*

Cannabinoid receptor agonists are considered to be a unique group for treating DPN. Nabilone, a synthetic cannabinoid (CB1 predominate) receptor agonist, effectively reduced DPN symptoms, improved sleep disruption, QOL, and patient status in general and enhanced patient satisfaction, nevertheless its effect on the patient’s physical and psychologic function was vague (136). On the other hand, the N-acylethanolamine fatty acid amide, palmitoylethanolamide, has potent analgesic, anti-inflammatory, and neuroprotective properties. Despite having little to no affinity for these receptors, it enhances anandamide’s activity at cannabinoid CB1 and CB2 receptors as well as TRPV1. The pain symptoms linked to DN were decreased after palmitoylethanolamide therapy (137). 

*Acetylcholine receptor agonists*

Nicotinic acetylcholine receptor-targeting substances have anti-nociceptive properties. Tebanicline, a strong nicotinic acetylcholine receptor agonist, has shown analgesic properties in a variety of nociceptive and neuropathic pain in preclinical models. A-366833, another nicotinic acetylcholine receptor agonist, lowers the mechanical hyperalgesia and anti-nociceptive activity in DM-induced neuropathic rat models (120, 138). It demonstrated considerably improved selectivity of the nicotinic acetylcholine receptor α4β2 subunit over the α3 subunit, thus this suggests a reduction in DPN pain without concomitant side effects, despite the fact that they have not as yet been examined clinically (139).

*Purinergic receptor blockers*

Excitatory P2X3 and P2X2/3 ATP-gated receptor channels directly sensitize C-fibers in response to membrane depolarization leading to calcium entry as well as purinergic signaling dysregulation resulting in pathological pain such as allodynia in DM (140). These receptors are linked to a higher pain score in DN patients (140, 141). It is interesting to know that this was demonstrated in female patients but not in male ones, suggesting a sex-specific mechanism for P2X7 receptor involvement in pain. A-317491 is the first non-nucleotide blocker for P2X3 homomeric and P2X2/3 heteromeric channels with very high affinity and selectivity for neuropathic pain. It has been found to be anti-nociceptive in rat models of chronic inflammatory and neuropathic pain, although, human studies are still missing (142, 143).

Sinomenine, an inhibitor of P2X3 agonist ATP-activated channels, reduced the hyperalgesia that type II DM rats experience and decreased the expression and activation of the P2X3 receptor (144, 145). It decreased the phosphorylation and activation of P38MAPK in type II DM DRG. Consequently, sinomenine administration may reduce P2X3 receptor expression and activation that have been up-regulated as well as the hyperalgesia that P38MAPK activation has exacerbated in type II DM rats (144).

*Angiotensin-2 receptor antagonist*

Both angiotensin-1 receptor (AT1R) and angiotensin-2 receptor (AT2R) were discovered in the CNS in the 1990s, which suggested using this system to manage pain. AT2R antagonists were discovered to have promising use for the treatment and identification of several neurological illnesses. Preclinical experiments conducted in 2021 supported the use of AT2R antagonists in neuropathic pain (146). Human peripheral somatic and visceral nerves express AT2 receptors, which co-localize with TRPV1 receptors to some extent. A highly selective AT2R antagonist, EMA401, can be used to decrease the increased TRPV1 ion channel sensitivity that is linked to the application of angiotensin II (147). The efficacy and safety of the medication, 400 mg twice daily, were established in a pilot phase IIa research. After 28 days of treatment, EMA401 significantly reduces postherpetic neuralgia symptoms compared to placebo (148). In 2021, EMA401 was studied in two phase IIb studies for its analgesic efficacy and safety in patients with severe DPN. These were multicenter, randomized, double-blind therapy studies carried out on patients with painful distal symmetrical sensorimotor neuropathy caused by type I/II DM. The main result for all studies was a significant reduction in the pain score (149).

*Sodium channel (v1.7 subtype-specific) blockers*

Three sodium channel subtypes, Nav1.7, Nav1.8, and Nav1.9, are more frequently linked to peripheral neurons than central neurons. These subtypes are crucial targets for pain relief due to their high expression in the somatosensory system, namely in neurons linked to DPN, and it has been documented that mutations in Nav1.7 are associated with painful hereditary diseases (150). PF**-**05089771, a selective peripheral Nav1.7 sodium channel blocker, has been studied against pDPN whereas it was administered at a dose of 150 mg twice daily and had a significant impact on pain scores yet less than the effect exhibited by pregabalin (151). 

*Miscellaneous pathways inhibition*

The abnormalities in Na/KATPase activity and NCV that are observed in DM patients are modulated by therapeutic treatment with PKF275-055, a long-acting new DPP-IV inhibitor. The PKF275-055 therapy gradually improves changes in heat responsiveness while raising mechanical sensitivity thresholds by about 50%. The anabolic impact of PKF275-055 enhances oral glucose tolerance and prevents the changes in Na/K-ATPase activity, NCV, and nociceptive thresholds observed in STZ-induced diabetic rats (152). Furthermore, researchers have proposed that therapies that target CXCL12/CXCR4 signaling may be a novel strategy for the treatment of pDPN. This is being explored in preclinical research, as pDPN symptoms including mechanical allodynia are related to chemokine CXCL12/CXCR4 signaling (153).

*Humanized monoclonal antibody*

Tanezumab, a novel treatment for neuropathic pain, is a fully humanized anti-NGF immunoglobulin G type II monoclonal antibody that is considered to be highly selective and specific for nerve NGF (154). Thus, it halts the binding between NGF and its receptors by firmly attaching to NGF, in that way disrupting pain signaling. Tanezumab dosages of 20 mg SC given on days 1 and 8 of treatment demonstrated a decrease in neuropathic pain (155). It has been observed that tanezumab was effective in DPN as it affects the longest axons in the peripheral nerve, which involves small fibers that are affected the most in DM (154). 

*Growth factors*

Nerve growth factor (NGF) is tightly related to the formation and growth of nerves. NGF concentrations that are abnormally high or low can harm neurons severely, since they have an impact on numerous survival pathways. Other substances that are related to proliferation, angiogenesis, sensitization, and cell growth include glia cell-derived neurotrophic factor, neurotrophic (NT-3, NT-4, and NT-5), brain-derived neurotrophic factor, as well as insulin growth factor I and II. The development of sympathetic neurons and tiny nerve fibers is regulated by NGF (156). 

Furthermore, evidence shows that decreased NGF availability may play a major role in the pathogenesis of DN, and animal models of neuropathy respond to the exogenous administration of NGF. Recombinant human NGF injection appeared to be beneficial in reducing the symptoms connected with DPN, according to two sets of phase II clinical trials (157). Moreover, the development of sympathetic neurons and small nerve fibers is regulated by NGF. Hyperglycemia affects retrograde axonal transport, NGF-dependent sensory neurons with reduced expression of neuropeptide substance P, a modulator of pain perception (18, 158). 

Interestingly, hepatocyte growth factor is expressed after a plasmid DNA injection into muscles, which suppresses proteins associated with pain in DRG neurons and subsequently spinal cord glial activation. Patients with DPN who received two doses of a plasmid containing HGF (VM202; 8 or 16 mg) intramuscularly demonstrated a substantial decrease in pain scores (159). 

*Topical agents*

Topical analgesic agents for pDPN include lidocaine, capsaicin, and nitrates, as well as intradermal injection of botulinum toxin-A. Topical lidocaine (5% patch or plaster; recommended by the AAN) successfully ameliorates the pain intensity in DN by its antagonistic effect on the sodium-gated voltage channels, i.e., Nav 1.7 and Nav 1.8 (160). The 5% lidocaine patch has a higher safety profile than pregabalin, but meta-analysis and systematic review studies have indicated that it has a comparable ability to reduce pain (161). Likewise, capsaicin, a TRPV1 receptor desensitizer, causes pain relief due to the release of substance P at nerve terminals, although it is known to cause degeneration of small nerve fibers. Capsaicin is FDA-approved only for foot pain relief (160). A three-month 8% capsaicin single application patch or a multiple daily application of capsaicin cream for several weeks results in adequate analgesia as compared to a placebo (162). 

On the other hand, topical nitrates for treating DPN are not recommended in any of the guidelines, however, they are used off-label (160). However, isosorbide dinitrate spray has shown promising outcomes in decreasing the burning sensation as well as the overall neuropathic symptoms in a randomized, placebo-controlled, double-blind study (163). Finally, botulinum toxin-A is utilized for DPN patients to minimize the symptoms of neuropathic pain and elevate mood by preventing acetylcholine release at the neuromuscular junction and regulating the firing of afferent sensory fibers when injected intra-dermally (164).

*Methadone*

Methadone is a synthetic opioid that has strong analgesic properties. Although it is commonly connected with the treatment of opioid addiction, licensed family physicians may prescribe it for analgesia. Methadone’s distinct pharmacokinetics and pharmacodynamics make it an important treatment choice for cancer pain and other chronic pain conditions, including neuropathic pain (165). Methadone metabolism and reaction differ from patient to patient. The transition to methadone and dosage titration should be done gradually and often monitored. Methadone is less expensive than other opioid formulations with continuous release (166).

***Non-pharmacological agents***

Along with pharmacological agents, the most significant interventional therapy for individuals with refractory neuropathic pain includes spinal cord stimulation (SCS) and physical activity (18).

**Spinal cord stimulation**

A low-voltage electric current is used to activate the dorsal columns of the spinal cord during spinal cord stimulation, an invasive method of treating chronic pain. Although the exact mechanism of action is yet unclear, it is believed that this intervention affects both the spinal and supraspinal regions. Six months of SCS treatment for DPN patients was proven to improve their pain symptoms and QOL (167). In accordance with results from a different recent study, this intervention was successful in DPN patients (168). In 2021, the FDA granted premarket approval to an implanted spinal cord stimulator for treatment of chronic pain associated with painful DN (https://practicalneurology.com/news/fda-approves-implanted-spinal-cord-stimulator-for-chronic-painful-diabetic-neuropathy; accessed 28/8/2022).

**Exercise**

Physical exercise was found to increase heat shock protein 72 (Hsp72) levels, which in turn reduced DPN symptoms in diabetic rats (63). Through exercise or manual techniques, the structure of the nervous system is mobilized by neurodynamics, also known as neural mobilization, thus restoring the nervous system’s structural balance. Numerous preclinical and clinical investigations have shown how well this intervention works to treat intraneural edema, thermal and mechanical hyperalgesia, and to restore fluid dispersion inside the neuron and immune response (169). It was reported that neural mobilization reduced levels of TNF-α and IL-1beta, which alleviated the mechanical allodynia in STZ-induced diabetic rats (170).

***Glycemic control***

Reduced HbA1c levels can ameliorate defects in vibration threshold, nerve conduction, and peripheral small nerve fiber function. Most DPN patients should aim for a HbA1c of less than 7% and clinicians should consider the dangers of hypoglycemia and a shorter life expectancy with relatively strict goals (171). Casadei *et al.* suggest that the QOL of patients could be greatly improved by comprehensive glycemic management by lowering the risk of ulcers and the number of future limb amputations and minimizing DM-related foot problems (172).

In DM patients, aggressive treatment may stop DPN from occurring. Evidence even points to the existence of a “metabolic memory” in patients who have previously undergone stringent glycemic management, which may be crucial in preventing the onset of DPN (173). However, intensive treatments like metformin and thiazolidinediones, were used in the BARI 2D experiment and considerably decreased the incidence of DPN (174).

Schematic diagram presenting the involvement of metabolic pathways; polyol, hexosamine, PARP, AGEs, and PKC pathways as well as inflammation and oxidative stress in the pathogenesis of DPN as a novel therapeutic target for treatment

Schematic diagram presenting the therapeutic agents targeting polyol, hexosamine, PARP, AGEs, and PKC pathways as well as inflammation and oxidative stress in the pathogenesis of DPN

## Conclusion

The development and pathophysiology of DPN are mainly attributable to a variety of signaling mechanisms. However, a thorough understanding of the precise modulation and particular role of the signal molecules is still missing. For that reason, finding a definitive cure for this persistent problem is challenging. The main therapeutic objectives of DN are slowing the developing progression and preventing its symptomatic consequences. However, the only strategy to treat DPN is to regulate its pathogenesis by activation of signaling pathways that repress neuropathy or inhibition of signaling pathways that promote it. This strategy has been shown to be effective in inhibiting pathogenic signaling in DPN in experimental studies as well as in various clinical trials. Tapentadol, an already-approved opioid receptor agonist-NE reuptake inhibitor, SNRIs, and cannabinoid receptor agonists are among novel agents that have shown promising outcomes for treating DPN. However, these agents must first undergo more extensive clinical trials and regulatory approvals before their use in DPN is recommended. 

## Future Recommendations

Numerous prospective targets in the management of DPN have been identified as a result of scientific developments in the field of the pathobiology of disease. These targets have been and can still be the focus of drug discovery efforts. The majority of these therapeutic targets, meanwhile, have yet to be investigated. Targeting them separately could not have enough of a clinical impact because several of these therapeutic targets seem to be interrelated. Research that combines these several possible targets could be successful. Such efforts will eventually aid in further easing some of the serious medical issues associated with treating DPN. Future research may reveal novel treatment targets and pharmacotherapeutic drugs if it focuses on better understanding the pathogenesis of DPN as well as pDPN.

Combining all of this evidence, the current review suggests additional studies are conducted to gain a better knowledge of the molecular processes involved in DPN, identify particular targets, and develop inhibitors and promoters of the target(s) as novel therapeutic approaches.

## Authors’ Contributions

 DO S designed the study. AA S collected data and drafted the manuscript. DO S and AA S approved the final version to be published.

## Conflicts of Interest

The authors declare no conflicts of interest exist.
